# Comparative In Vitro Evaluation of Buccal Films, Microcapsules, and Liposomal Systems for Naringin and *Citrus × paradisi* L. Peel Extract: Effects of Encapsulation Strategy and Compound Origin on Release Profiles

**DOI:** 10.3390/pharmaceutics17101311

**Published:** 2025-10-09

**Authors:** Jolita Stabrauskiene, Mindaugas Marksa, Jurga Bernatoniene

**Affiliations:** 1 Department of Drug Technology and Social Pharmacy, Lithuanian University of Health Sciences, LT-50161 Kaunas, Lithuania; jolita.stabrauskiene@lsmu.lt; 2 Institute of Pharmaceutical Technologies, Lithuanian University of Health Sciences, LT-50161 Kaunas, Lithuania; 3 Department of Analytical and Toxicological Chemistry, Lithuanian University of Health Sciences, LT-50161 Kaunas, Lithuania; mindaugas.marksa@lsmuni.lt

**Keywords:** naringin, grapefruit peel extract, liposomes, microencapsulation, buccal films, dissolution, mucoadhesion

## Abstract

**Background/Objectives**: *Citrus × paradisi* Macfad., Rutaceae. peel is a rich source of naringin (NR), but its poor solubility and low bioavailability limit applications. This study aimed to improve NR delivery by comparing microencapsulation, liposomal microencapsulation, and buccal films containing either pure NR or grapefruit peel extract. **Methods**: Four spray-dried powder formulations—spray-dried NR (NS), liposomal NR (NLS), spray-dried extract (ES), and liposomal extract (ELS)—were produced using maltodextrin, β-cyclodextrin, and HPMC as wall materials. Buccal films (EP1, EP2, NP1, NP2) were prepared via solvent casting with HPMC, alginate (ALG), or polyvinyl alcohol (PVA). All samples were evaluated for solubility, moisture content, mucoadhesion, and in vitro release under simulated gastric, intestinal, and salivary conditions. **Results**: NR powders had the highest absolute solubility (306.42 ± 10.34 µg/mL), whereas ELS showed the lowest due to low loading. However, relative to theoretical NR content, ELS achieved the highest dissolution efficiency (55.3%), followed by NLS (42.7%), outperforming NS (5.6%) and ES (91.8%) in sustained release potential. Dual encapsulation (NLS, ELS) slowed gastric release and maintained intestinal delivery, while non-liposomal powders released rapidly. In buccal films, NP2 (NR + PVA) showed the highest release (69.97 ± 3.01 µg/mL; 40.9% efficiency) and strongest mucoadhesion (0.47 N·s). Extract-based films had lower absolute NR release but higher relative efficiency to content, likely due to co-extracted compounds enhancing wettability and matrix erosion. **Conclusions**: Liposomal microencapsulation improves relative dissolution efficiency and sustains intestinal release, while PVA-based buccal films enhance both release and mucoadhesion. Polymer choice and active ingredient composition are critical for optimising oral delivery of NR. These results demonstrate the potential of the proposed systems in the pharmaceutical or dietary supplement field, especially in improving the oral delivery of poorly soluble flavonoids. A graphical summary is included, visually summarising the main formulation strategies and results.

## 1. Introduction

Grapefruit (*Citrus × paradisi* Macfad., Rutaceae) by-products have recently attracted growing interest because of their high content of health-promoting phytochemicals. Among these, naringin (NR) and its aglycone naringenin (NAR) are the most important compounds. They are known for their antioxidant, anti-inflammatory, and anticancer properties [1,2,3,4]. Beyond naringin, grapefruit peel extract also contains flavonoid glycosides, organic acids, and natural sugars, which exhibit additional antioxidant, antimicrobial, and permeability-enhancing effects, potentially offering synergistic therapeutic or functional benefits in oral delivery systems.

However, both molecules face a common challenge: they are poorly soluble in water and have very low bioavailability in the human body. For example, naringin dissolves only at approximately 38 μg/mL and exhibits less than 5% oral bioavailability, classifying it as a BCS class IV [5,6,7,8]. These limitations reduce its effectiveness when used in foods, nutraceuticals, or therapeutic applications.

Different encapsulation technologies are being explored to address these problems. Microencapsulation, typically carried out with carriers such as maltodextrin or β-cyclodextrin, allows better stabilisation of NR and provides controlled release. In our previous work on the encapsulation of *C. paradisi* peel extracts, we compared different carrier compositions. We demonstrated that the combination of maltodextrin, β-cyclodextrin, and carboxymethylcellulose provided the most favourable results, achieving high encapsulation efficiency (>90%), improved solubility, and stable powder characteristics. These findings indicated that polysaccharides and cyclic oligosaccharides act synergistically to stabilise flavonoids and enhance their release properties [9]. Liposomes, which are vesicles formed from phospholipid bilayers, can further increase solubility and protect hydrophobic molecules such as NR [10,11,12,13]. Since liposomal dispersions in water are often unstable, they are commonly converted into dry powders through lyophilisation or spray drying. A promising strategy is dual encapsulation, where liposomes are trapped inside carbohydrate-based microcapsules to reduce release in the acidic stomach environment while supporting prolonged release in the intestine. Although this method has not yet been suggested, notable improvements in solubility and stability have been observed when directly applied to naringin [14,15,16].

Another innovative delivery method is the use of buccal films. These thin, mucoadhesive formulations adhere to the buccal mucosa, allowing drugs to be absorbed directly into the bloodstream and bypassing first-pass metabolism in the liver. Their swelling properties and polymer composition have a significant impact on the rate of release of the active compound. Studies have shown that selecting the polymer matrix can significantly improve the release and absorption profiles [17,18].

Summarising these limitations, it becomes evident that a systematic comparison of different encapsulation strategies is required. Therefore, this study compares three delivery paths for *C. paradisi*. peel extract and pure NR: (i) microencapsulated powder, (ii) spray-dried liposomal powder, and (iii) buccal films. All were evaluated in vitro for solubility, stability, and release under simulated gastric (pH ~1.2), intestinal (pH ~6.8), and artificial saliva (pH ~6.8) conditions.

## 2. Materials and Methods

Fresh grapefruit peels were collected post-juicing, dried at 60 ± 5 °C, ground, and stored in a dry, dark place. Naringin (≥98% purity) was obtained from Sigma-Aldrich (Buchs, Switzerland). Lipoid S100 and methanol (99%) were sourced from Lipoid GmbH and Carl Roth GmbH, respectively (Germany). Ethanol (96%) was sourced from Vilniaus Degtinė (Vilnius, Lithuania). Purified water was prepared using a Millipore system (Merck, Rahway, NJ, USA). Hydroxypropyl methylcellulose (HPMC, Methocel E5 Premium LV) was obtained from Dow Chemical Co. (Midland, MI, USA). Sodium alginate (Protanal^®^ LF 10/60, ALG) was sourced from FMC BioPolymer (Philadelphia, PA, USA). Polyvinyl alcohol (PVA) and glycerol (≥99.5%) were obtained from Sigma-Aldrich (St. Louis, MO, USA). Additional reagents for artificial saliva, simulated gastric fluid (SGF), and simulated intestinal fluid (SIF) were sourced from Sigma-Aldrich, Merck, and Farmalabor.

### 2.1. Preparation of Extract

**Figure 1:**
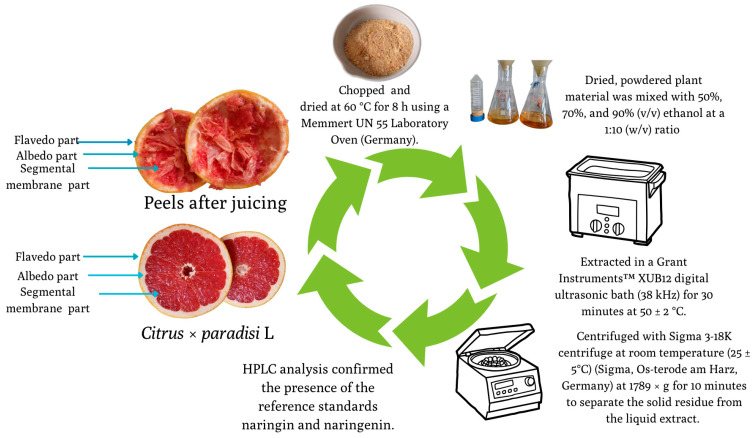
Extraction procedure of bioactive compounds from *C. paradisi* peels, including drying, ethanol extraction, ultrasonic treatment, centrifugation, and HPLC analysis.

Dried peel powder was mixed with 50%, 70%, or 90% ethanol (*v*/*v*) at a 1:10 (*w*/*v*) ratio and subjected to ultrasonic-assisted extraction (38 kHz, 30 min, 50 ± 2 °C). Extracts were cooled, centrifuged (1789× *g*, 10 min), filtered (0.22 µm PVDF membrane), and stored for further use. The process is demonstrated in Figure 1. The ethanol concentrations used for extraction (50%, 70%, and 90%) were based on our prior optimisation studies, which evaluated the influence of solvent strength on flavonoid yield and formulation compatibility [19,20].

Depending on the specific requirements of each formulation and the solubility properties of the active compounds, concentrations of 50%, 70%, and 90% ethanol were selected. For liposomal formulations, 90% ethanol was used to ensure efficient dissolution of the lipid components (Lipoid S100 and cholesterol) together with the active ingredients. For spray-dried formulations, 50% ethanol was chosen due to its favourable interaction with wall materials and its ability to form stable emulsions. For buccal film formulations, 70% ethanol ensured an optimal balance between extract solubility and compatibility with film-forming polymers, such as HPMC, ALG, and PVA, during the solvent casting process.

### 2.2. HPLC Methodology for the Quantification of Naringin and Naringenin

**Figure 2:**
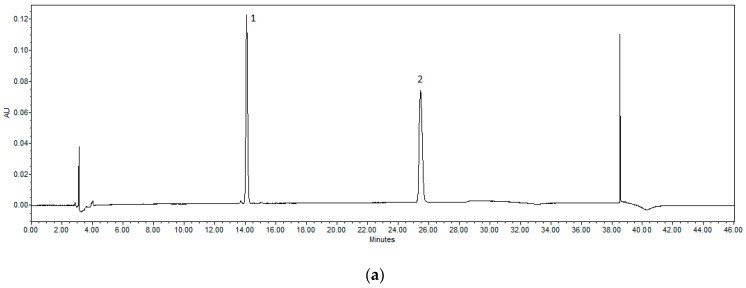

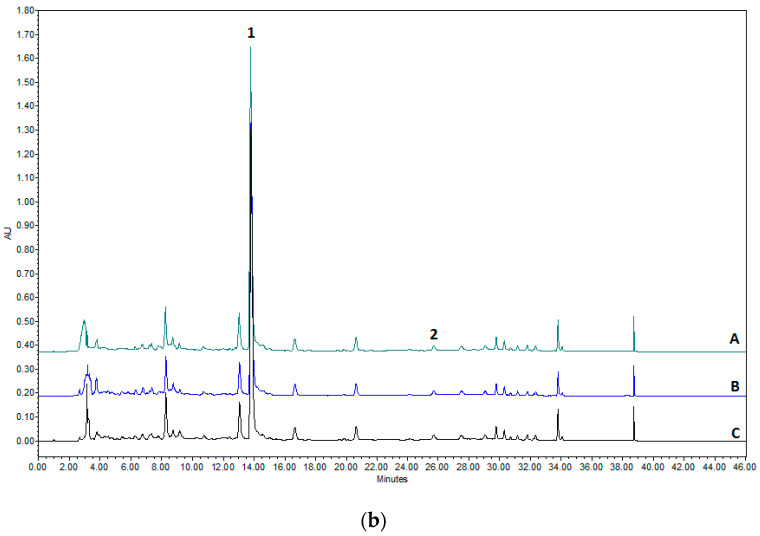
(**a**). Chromatograms of standards detected by HPLC. Peaks identified: 1—naringin; 2—naringenin. (**b**). HPLC chromatogram of the *C. paradisi* peel extract obtained using ethanol as extraction solvent at different concentrations: A—50%, B—70%, and C—90%. Peaks corresponding to naringin—1 and naringenin—2 are indicated. All experiments were conducted in triplicate (*n* = 3).

The study employed a Waters 2695 liquid chromatography system with a photodiode array detector (Waters 996, wavelength range 200–400 nm) to analyse biologically active compounds. The chromatographic separation was achieved using an ACE C18 column (250 mm × 4.6 mm, 5 μm particle size) with a gradient elution method. A total of 10 μL of each extract was injected and analysed at a wavelength of 280 nm. A mixture of acetonitrile (A) and water (B) was employed as the mobile phase, pumped at a constant flow of 1 mL/min: at 0.0 min, 10% A; at 5 min, 20% A; at 25 min, 40% A; at 30 min, 100% A; at 35 min, 100% A; and returning to 10% A at 36 min. The column temperature was maintained at 25 °C. Peak identification was performed by comparing the UV-vis spectra and retention times of the compounds to those of authentic reference standards (Figure 2). Each sample was analysed in duplicate. A 100 μg/mL solution of naringin and naringenin was dissolved in 70% methanol to serve as the reference standard. From this, a series of six concentrations was prepared to generate calibration curves. Each concentration was injected three times to assess linearity. The calibration equations for naringin and naringenin were derived from plotting their peak areas against their respective concentrations, yielding regression coefficients (R^2^) greater than 0.999, indicating excellent linearity. The method’s sensitivity was assessed by determining the limit of detection (LOD) and limit of quantitation (LOQ), calculated based on signal-to-noise ratios of 3 and 10, respectively. Intra-day and inter-day precision were evaluated using a standard mixture of naringin and naringenin, with five consecutive injections performed on the same day over four different days. Results were expressed as relative standard deviation (RSD). The study confirmed the retention times and spectra of naringin and naringenin against the prepared extracts. The linearity of the calibration curves was established, with naringin exhibiting a linearity range of 1.166 to 33.343 μg/mL and naringenin from 0.472 to 15.125 μg/mL. Quantification results were reported in μg/g and mg/g dry weight (DW) for naringenin and naringin, respectively [21].

### 2.3. Preparation of C. paradisi Peel Extracts and Naringin-Loaded Liposomes

Liposomes were prepared by ethanol injections followed by probe sonication. Lipoid S100 and cholesterol were dissolved in 10 mL of 90% ethanolic solution containing either grapefruit peel extract or pure NR (10 mg/mL), then injected into the aqueous phase under constant stirring.

**Figure 3:**
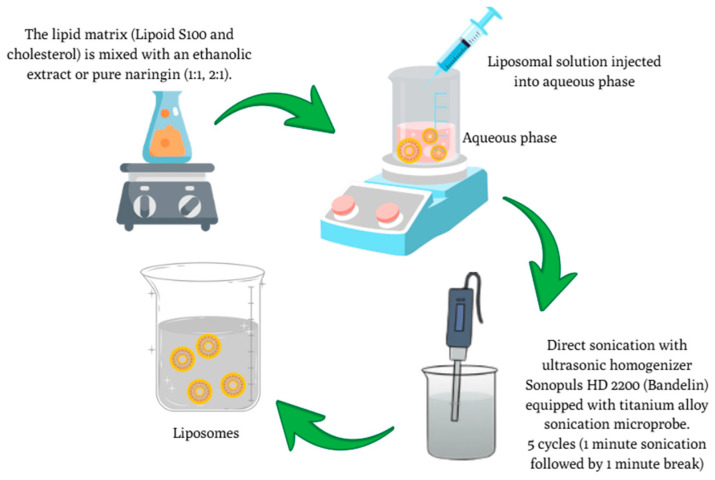
Schematic representation of the liposome preparation process using lipid matrix, aqueous phase injection, and ultrasonic homogenization.

Sonication (5 cycles, 1 min on/1 min off, 16 ± 5% power) produced nanoscale vesicles, as represented in Figure 3. Formulations varied in lipid-to-core ratio (1:1 and 2:1) as described in Table 1. Liposomes were prepared according to a modified procedure based on the method reported by San Ang et al. [22].

### 2.4. Characterisation of Particle Size Distribution and Zeta Potential

The particle size and size distribution of the liposomal formulations were determined using dynamic light scattering (DLS) with a Nano ZS 3600 system (Malvern Instruments, Worcestershire, UK). Measurements provided both the mean hydrodynamic diameter and the polydispersity index (PDI).

Zeta potential was measured on the same instrument in zeta mode. For these measurements, the samples were placed in a dedicated cell equipped with electrodes, which ensured the generation of a stable electric field and improved the precision of the readings. The procedure followed the approach described by Németh et al. [23].

### 2.5. Spray-Drying Microencapsulation of C. paradisi Peel Extracts and NR Samples

Spray-drying conditions and wall material ratios were chosen based on previously optimised protocols developed in our earlier studies [9]. Based on our previous research, four different samples were prepared for microencapsulation using spray-drying: (1) *C. paradisi*. peel extract (ES), (2) pure NR solution (50 mg/mL) (NS), (3) liposomal *C. paradisi* peel extract (ELS), and (4) liposomal NR solution (10 mg/mL) (NLS).

The encapsulation matrix was prepared by dissolving maltodextrin (MD, 15% *w*/*v*), β-cyclodextrin (β-CD, 2.5% *w*/*v*), and carboxymethylcellulose sodium salt (HPMC, 0.5% *w*/*v*) in purified water. After the complete dissolution, each active ingredient was homogenised with the wall material solution to form stable emulsions.

**Figure 4:**
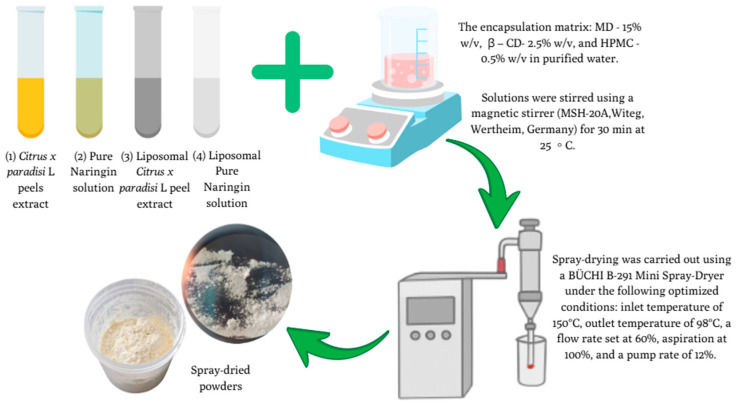
Schematic illustration of the microencapsulation process of different samples using spray-drying technology under optimised conditions. Sample ID: 1—ES, 2—NS, 3—ELS, 4—NLS.

Spray-drying was carried out using a BÜCHI B-291 Mini Spray-Dryer under the following optimised conditions: inlet temperature of 150 °C, outlet temperature of 98 °C, a flow rate set at 60%, aspiration at 100%, and a pump rate of 12%, as demonstrated in Figure 4. The resulting spray-dried powders were carefully collected and stored at +4–7 °C to preserve their stability and functional properties for further analysis [23,24].

### 2.6. SEM Analysis of Microcapsules: Morphological Evaluation

The surface morphology of spray-dried microcapsules was evaluated using a Hitachi TM 3000 SEM (Tokyo, Japan) at 100×–6000× magnification and 5 kV accelerating voltage. Samples were mounted on stubs using double-sided tape for imaging [25]. All experiments were conducted in triplicate (*n* = 3)

### 2.7. Encapsulation Efficiency (EE)

Encapsulation efficiency (EE) of the liposomes was evaluated by centrifuging the formulations at 10,000× *g* for 30 min. The supernatant containing the unencapsulated active compounds was carefully extracted [26]. All experiments were conducted in triplicate (*n* = 3).

The supernatant was analysed using HPLC to quantify the concentration of unencapsulated active compounds. The EE of NR were calculated using the formulation:

(1) $$EE\;(\%)=\frac{QT-Q_{FREE}}{QT}\times 100$$

*QT* represents the total active compound added (mg/g), *QFREE* denotes the non-encapsulated fraction in the supernatant (mg/g), and *EE* (%) indicates the encapsulation efficiency in the final powder.

### 2.8. Characterisation of Spray-Dried Powders

#### 2.8.1. Yield Calculation

The powder yield obtained from the spray-drying process was calculated as the ratio between the total mass of dry powder collected and the total amount of solid raw materials initially present in the feed solution [27].

(2) $$Yield\;\left(\%\right)=\frac{Total\;weight\;of\;dry\;powdrer\;collected}{Total\;weight\;of\;row\;materials\;in\;feed\;solution}\times 100\;$$

#### 2.8.2. Evaluation of Moisture Content in Spray-Dried Powders

Moisture content was determined using a Kern DBS60-3 moisture analyser (Kern & Sohn GmbH, Balingen, Germany). Approximately 1.0 ± 0.05 g of each sample was uniformly distributed on the weighing plate and analysed in triplicate. The mean value was calculated and expressed as a percentage of moisture content [28]. All experiments were conducted in triplicate (*n* = 3).

#### 2.8.3. Aqueous Solubility Determination of the Spray-Dried Powders

The theoretical naringin content (mg) was calculated from the known concentration in the formulation and the mass of the powder used (500 mg). The dissolved amount was quantified using HPLC as described above.

The aqueous solubility of the spray-dried powders was determined following a modified equilibrium solubility method [22]. A total of 0.5 g of each powder was dispersed in 30 mL of distilled water in sealed vials. Samples were shaken at 25 °C for 24 h at 120 rpm to reach equilibrium. After incubation, suspensions were centrifuged at 10,000 rpm for 10 min. The supernatants were collected and filtered through a 0.22 µm syringe filter. The concentration of dissolved compounds was determined using HPLC and expressed in µg/mL. All experiments were conducted in triplicate (*n* = 3).

#### 2.8.4. In Vitro Release Study of NR from Microcapsules and Liposomal Powders

The in vitro release study of the active compound, NR, from microcapsules and liposomal powder formulations was conducted using a Sotax AT7 Smart Dissolution System (SOTAX AG, Aesch, Switzerland). The experimental protocol was based on the previously reported methodology by Kazlauskaitė et al. [29], with minor modifications adapted for the tested formulations. Simulated gastric fluid (SGF, pH 1.2) was prepared in accordance with the European Pharmacopoeia by dissolving 2.0 g of NaCl, 3.2 g of pepsin, and 80 mL of 1 M HCl in distilled water, adjusting the final volume to 1000 mL. Simulated intestinal fluid (SIF) was prepared using 6.8 g of KH_2_PO_4_, 10 g of pancreatin, and 77.0 mL of 0.2 M NaOH, followed by dilution with distilled water to a final volume of 1000 mL.

Samples were first incubated in SGF for 0–90 min, followed by transfer to SIF for an additional 90–180 min, simulating gastrointestinal transit. Aliquots were collected every 30 min over a total release period of 0–180 min.

All collected samples were filtered through a 0.45 μm membrane filter and subsequently analysed using high-performance liquid chromatography (HPLC) for the quantitative detection of the flavanone naringin. All experiments were conducted in triplicate (*n* = 3).

### 2.9. Preparation of Buccal Films

Buccal films were obtained via solvent casting. Formulations EP1 and EP2 contained 70% ethanolic grapefruit peel extract, while NP1 and NP2 contained pure NR (50 mg/mL in 70% ethanol).

**Figure 5:**
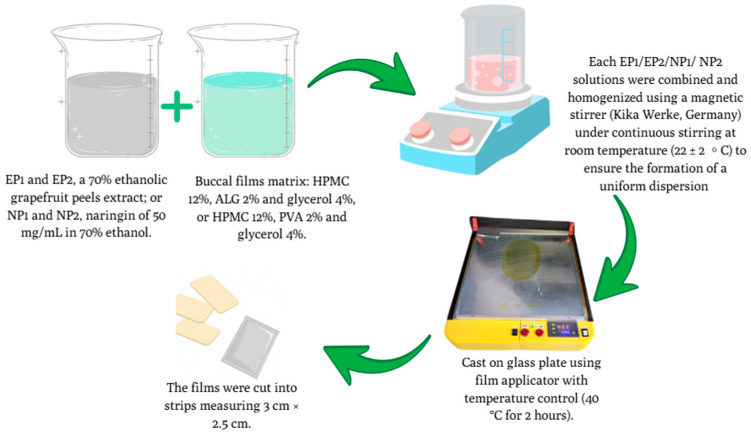
Schematic representation of buccal film preparation using the solvent casting method with naringin (NP1, NP2) or grapefruit peel extract (EP1, EP2), film-forming, drying, and cutting processes. All experiments were conducted in triplicate (*n* = 3).

A 12% (*w*/*v*) HPMC solution was prepared using a 70:30 mixture of NR or extract solution (in 70% ethanol) and purified water, ensuring complete polymer dispersion before blending with secondary components. Separately, sodium alginate (2% *w*/*v*) for EP1 and NP1, or PVA (2% *w*/*v*) for EP2 and NP2, was dissolved in glycerol (4% *w*/*v*). Solutions were combined, homogenised, and cast using a ZUA 2000 film applicator (2 mm wet thickness) onto glass plates. Films were dried at 40 °C for 2 h, cut into 3 cm × 2.5 cm strips, sealed in foil pouches, and stored at 22 ± 2 °C [30,31]. The compositions of the prepared buccal film formulations are summarised in Table 2. All processes are demonstrated in Figure 5.

#### 2.9.1. Texture Analysis of Buccal Films

Film mechanical properties were measured using a TA.XT Plus texture analyser (Stable Micro Systems Ltd., Godalming, UK), based on modified procedures from prior literature [32].

For mucoadhesion, 3 cm × 2.5 cm dry film samples were adhered to a fixed plastic support to determine adhesive strength. A transparent plastic ring with a 1 cm central opening (A/MUC mucoadhesive probe) was aligned over the sample, leaving an exposed circular area. This area was hydrated with 100 µL of artificial saliva (pH 6.8) and allowed to equilibrate for 15 s. A cylindrical P/0.5R probe (slightly smaller diameter than the opening) was then lowered onto the moistened surface under a constant load of 5 N for 60 s. The force required to detach the probe from the film (adhesive strength, in N) was measured. Each test was performed three times under room temperature conditions. All experiments were conducted in triplicate (*n* = 3).

#### 2.9.2. Buccal Film Moisture Content Determination

Film moisture content (%) was determined by drying to constant weight at 105 °C in an analyser (DBS 60-3, KERN & SOHN GmbH, Balingen, Germany), in triplicate [33,34]. All experiments were conducted in triplicate (*n* = 3).

#### 2.9.3. In Vitro Release Profile of NR from Buccal Films

The dissolution of mucoadhesive polymeric films (*n* = 3) was tested in 25 mL of artificial saliva (pH 6.8) at 37 ± 1 °C. Each 3 cm × 2.5 cm film was placed in a Berzelius beaker under static conditions. The time to complete dissolution, with no visible residue, was recorded using a method adapted from Y. Maslii et al. [34].

For NR release studies, 3 cm × 2.5 cm buccal films were placed in 25 mL of artificial saliva at 37 °C under constant stirring. The average film weights were as follows: EP1—108.17 mg, EP2—163.27 mg, NP1—97.64 mg, and NP2—97.72 mg. Aliquots (1 mL) were withdrawn at 5, 10, 15, and 30 min, stored, and analysed by HPLC. Artificial saliva was prepared by dissolving MgCl_2_ (100 mg/L), CaCl_2_·2H_2_O (220 mg/L), Na_2_HPO_4_·7H_2_O (1350 mg/L), KH_2_PO_4_ (680 mg/L), KCl (750 mg/L), urea (600 mg/L), and NaCl (600 mg/L) in distilled water and adjusting pH to 6.8 [34,35]. All experiments were conducted in triplicate (*n* = 3).

### 2.10. Statistical Analysis

All data are presented as mean ± standard deviation (SD) from three independent experiments. Statistical significance (*p* < 0.05) was assessed using one-way ANOVA, with post hoc non-parametric tests (Friedman, Wilcoxon, Mann–Whitney U) where appropriate. Correlation and regression were analysed using Spearman’s method. Analyses were performed using SPSS v20, GraphPad Prism 8, and Excel 2021.

## 3. Results and Discussion

### 3.1. Evaluation of Flavonoid Content in Hydroalcoholic Peel Extracts of C. paradisi

Quantitative HPLC analysis confirmed that NR was the predominant flavonoid in all hydroalcoholic extracts of *C. paradisi* peel, whereas NAR was only a minor component. The highest NR concentration was obtained in the 50% EtOH extract (15.62 mg/g), followed by the 90% extract (15.40 mg/g) and the 70% extract (14.58 mg/g). In contrast, NAR levels were low and relatively stable (1.41–1.82 µg/g), confirming its limited abundance in grapefruit peel.

These results indicate that solvent concentration in the tested range (50–90% ethanol) did not markedly affect NR yield, as the differences between extracts were within a narrow margin (<1.1 mg/g). Nevertheless, the slight superiority of the 50% EtOH extract suggests that moderate ethanol content may facilitate better solubilisation of glycosylated flavonoids such as NR.

Importantly, variability between studies is evident: while our extracts contained ~14–16 mg/g naringin, our previous work reported up to 42.04 mg/g [19,20]. This discrepancy is most likely due to natural differences in raw material (ripeness, harvest season, or cultivation conditions) rather than methodological inconsistencies [36].

### 3.2. Characterisation of Liposomal Formulations: Particle Size, PDI, and Zeta Potential

The physicochemical properties of the liposomal formulations, including particle size, polydispersity index (PDI), and zeta potential, are summarised in Table 3.

The particle size of the liposomes ranged from 93.93 ± 4.70 nm to 101.5 ± 5.08 nm, placing all formulations within the nanoscale range, which is favourable for biomedical applications such as drug delivery [37]. EL2 had the smallest particle size (93.93 ± 4.70 nm), which was significantly smaller than that of EL1 (101.5 ± 5.08 nm, *p* < 0.05). NL1 and NL2 showed intermediate sizes with no statistically significant difference from the extract-based systems EL1 or EL2. These findings suggest that the 2:1 lipid-to-core ratio promoted smaller vesicle formation, consistent with previous reports that higher lipid concentrations enhance bilayer stability and reduce vesicle size [38]. NL2 (96.96 ± 4.85 nm), which had the same lipid composition as EL2 but contained naringin instead of extract, showed slightly smaller particles than NL1 (98.57 ± 4.93 nm), further suggesting that lipid content—not the encapsulated compound—was the main factor causing size reduction [39,40].

PDI values ranged from 0.144 to 0.362. EL2 had the lowest PDI (0.144 ± 0.007), indicating a highly uniform particle size distribution. EL1 and NL1 had significantly higher PDI values, suggesting greater heterogeneity. Higher lipid content in lipid nanoparticles likely improves homogeneity by stabilising vesicle formation. This stabilisation is due to the ability of lipids to interact with each other and with other components of lipid nanoparticles, such as cholesterol, which promotes a more uniform structure and prevents aggregation or phase separation [41].

Zeta potential ranged from −10.4 mV (NL1) to −25.8 mV (NL2). NL2 showed the most negative value (−25.8 ± 1.29 mV), indicating superior electrostatic stability and reduced risk of aggregation. NL1 had the least negative zeta potential (−10.4 ± 0.52 mV), while EL1 and EL2 were intermediate. Similar correlations between increased lipid content and more negative zeta potential have been found by Németh et al. [23], suggesting that higher lipid levels enhance the surface charge density of the vesicles. Based on physicochemical evaluation, EL2 and NL2 were selected for further studies. These size values (<120 nm) and zeta potentials below −20 mV suggest good colloidal stability and favourable biological behaviour, potentially enhancing mucosal permeation and systemic absorption after oral administration [42,43].

### 3.3. Characterisation of Spray-Dried Microcapsules and Spray-Dried Liposomes

#### 3.3.1. Powder Yield, Moisture Content, and Encapsulation Efficiency

The spray-drying process produced powders with yields ranging from 36.7% to 43.0% (Table 4).

Extract-based powders (ES, ELS) yielded higher amounts (41–43%) than NR-based powders (NS, NLS; 36–38%, *p* < 0.05). This difference likely reflects the higher solid content and greater compatibility of the natural extract with the carrier, which promotes droplet stability and efficient solvent removal during the drying process. Similar effects have been observed in studies comparing plant extracts with pure compounds in capsule manufacturing processes [44]. Moreover, liposomal formulations (ELS and NLS) yielded slightly lower results compared to non-liposomal formulations. This reduction is likely due to the additional lipid phases, which can affect atomization and increase wall deposition during the spray drying process. Similar observations have been reported in previous studies, where lipid-rich formulations reduced powder recovery due to nozzle clogging and increased wall deposition [45,46]. These findings align with reports that high fat content can adversely affect the retention of bioactive compounds and the overall efficiency of encapsulation processes [47].

Moisture content was below the 6% stability threshold for all samples [27]. NLS had the highest residual moisture (5.58 ± 0.279%, *p* < 0.05). This result can be explained by the interaction between crystalline NR and the lipid bilayer, which may prevent the complete removal of bound water during the drying process. Cegledi et al. [48] published a similar study on flavonoids and lipids, noting that a possible interaction between polyphenols and lipids may increase the residual moisture content.

#### 3.3.2. Encapsulation Efficiency

Encapsulation efficiency (EE) showed apparent differences between the tested systems, ranging from 81.08% for the spray-dried NR sample (NS) to 99.36% for the extract-based liposomal powder (ELS). In general, liposomal systems (ELS, NLS) performed better than non-liposomal ones, supporting the well-known function of phospholipid bilayers in retaining both hydrophilic and lipophilic compounds within the carrier. The exceptionally high EE observed for ELS (99.36 ± 4.96%) suggests that the presence of multiple extract components may stabilise the liposomal structure and enhance the retention of NR during spray-drying. This result aligns with previous reports that complex plant matrices often interact more strongly with carrier materials than single, pure compounds, leading to improved entrapment and stability. The lower encapsulation efficiency observed for crystalline NR could also be attributed to partial precipitation or phase separation during spray drying, as previously observed in similar flavonoid systems where crystalline nature interfered with uniform entrapment [47,49,50].

#### 3.3.3. Solubility and Dissolution Efficiency

The aqueous solubility of NR was evaluated by measuring the concentration of the dissolved compound in 30 mL of distilled water from 500 mg of each spray-dried powder formulation. The results are summarised in Table 4.

The literature reports a wide range of solubility data for NR, ranging from 30–40 µg/mL under standard experimental conditions to a theoretical solubility of 0.5 g/L (500 µg/mL) at 20 °C and up to 1 mg/mL at 40 °C, confirming the significant influence of temperature [7]. The highest solubility was observed for the spray-dried pure NR formulation (NS), reaching 306.42 ± 10.34 µg/mL, nearly tenfold higher than the reported reference value. The extract-based microencapsulated powder (ES) also showed a significantly higher solubility at 138.80 ± 4.25 µg/mL. The solubility of the liposomal naringin formulation (NLS) reached 93.32 ± 6.01 µg/mL, more than twice the solubility of pure crystalline naringin. Meanwhile, liposomal grapefruit peel extract powder (ELS) had the lowest solubility among all tested samples—17.36 ± 1.01 µg/mL.

The increasing solubility observed in NS, ES, and NLS formulations can be attributed to the spray-drying process, combined with the use of appropriate carrier materials, which enhance wettability, reduce particle crystallinity, and increase surface area. Meanwhile, the low solubility of the ELS formulation, despite its high encapsulation efficiency (99.36 ± 4.96%), can be attributed to several factors. First, the initial amount of extract used in the formulation was relatively low, resulting in a limited absolute NR content in the final dried product (0.94 mg of theoretical NR in 500 mg of powder), as shown in Table 5. Second, liposomal encapsulation may limit the release of NR due to entrapment in lipid bilayers, especially when drug loading is low. Lastly, matrix entrapment and reduced wettability of lipid-rich particles may delay the diffusion of NR [48,50].

Meanwhile, these findings suggest that while liposomal systems, such as NLS, are valuable for enhancing NR stability and controlling its release, their apparent solubility may be lower than that of non-liposomal powders. This finding is compatible with previous studies, which have shown that liposomal carriers tend to slow down the initial dissolution phase while providing benefits for sustained release and improved absorption in vivo [6,51,52,53].

#### 3.3.4. Theoretical and Dissolved Amounts of NR

To ensure clarity, dissolution data were expressed both as absolute NR concentrations and as dissolution efficiencies relative to the theoretical drug content. This approach enables a more direct comparison of different formulations, highlighting the higher relative efficiency of dual encapsulation (NLS, ELS) compared to single microencapsulation (NS, ES).

The spray-dried NR powder (NS) contained the highest theoretical amount of NR (163.82 ± 2.10 mg), but released only 9.19 ± 0.31 mg, corresponding to a very low DE of 5.6%. This low percentage demonstrates the limitations of the solubility and slow dissolution kinetics of crystalline NR, even after encapsulation with maltodextrin, β-cyclodextrin, and HPMC. Similar limitations have been reported for flavonoids encapsulated in carbohydrate-based matrices [51], where strong intermolecular hydrogen bonding, high crystallinity, and limited wettability of the carrier system hinder complete dissolution.

In contrast, the NLS formulation with a much lower drug loading (6.55 ± 0.22 mg) achieved a DE of 42.7 ± 2.13%, indicating more efficient release relative to the available content. This suggests that liposomal encapsulation can increase the relative release efficiency even if the total amount of solute remains lower.

For the extract-based systems, ES achieved the highest DE among all tested samples (91.8 ± 4.59%), releasing nearly all its naringin content (4.16 ± 0.12 mg of 4.53 ± 0.15 mg theoretical). The presence of other phytochemicals and soluble matrix components in the extract may have enhanced solubilisation, acting as natural surfactants and dissolution promoters [49].

Interestingly, ELS, despite having the lowest absolute naringin loading (0.94 ± 0.05 mg), achieved a DE of 55.3 ± 2.76%. While lower than ES, this still demonstrates that more than half of the encapsulated naringin was released into the medium. This suggests that lipid encapsulation, even in low-loading systems, can sustain and control the release rate, reducing burst dissolution and potentially enhancing bioavailability in vivo [5,54].

Overall, the data indicate that while single-layer carbohydrate encapsulation (NS) offers some protection to NR, it does not overcome its intrinsic solubility limitations. Incorporating a lipid phase, as in NLS and ELS, markedly improves relative dissolution efficiency, although achieving high solubility still requires optimising both drug loading and release characteristics.

#### 3.3.5. Scanning Electron Microscopy of Spray-Dried Powder

**Figure 6:**
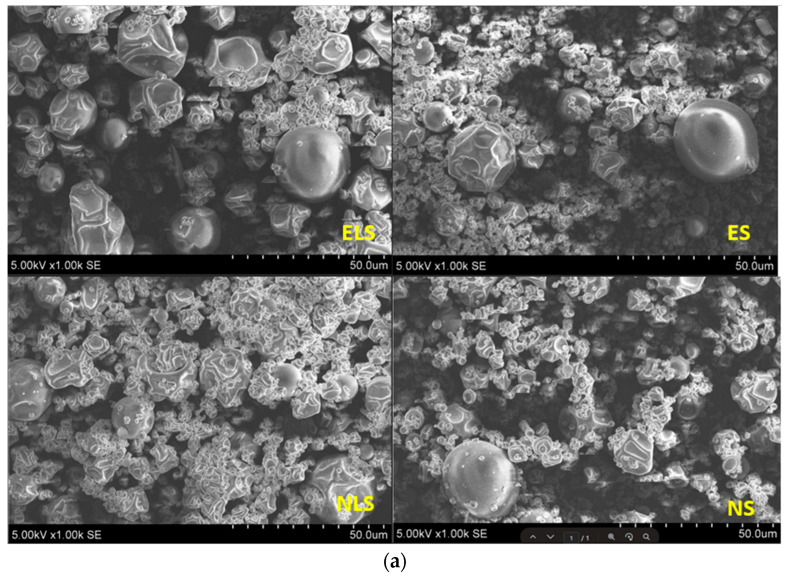

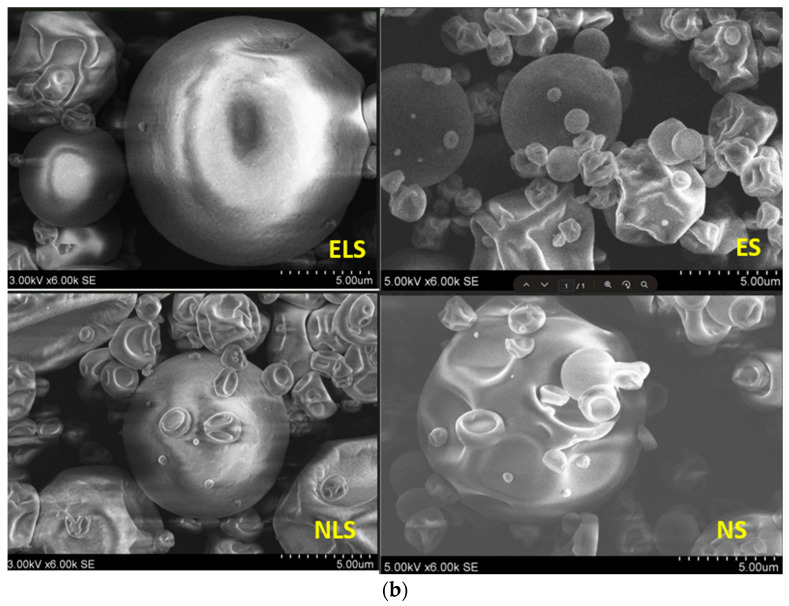
(**a**) SEM images of spray-dried powders (ELS, ES, NLS, NS) at ×1000 magnification. (**b**) SEM images of spray-dried powders (ELS, ES, NLS, NS) at ×6000 magnification. Quantitative image analysis of SEM micrographs (×6000 magnification) revealed a predominant particle size range below 1 µm, with a mean equivalent diameter of 1.45 µm, supporting the uniformity and nanoscale nature of the spray-dried formulations.

The surface morphology of the spray-dried formulations was examined by scanning electron microscopy (SEM) at magnifications of ×1000 and ×6000 (Figure 6). All samples were prepared using an encapsulation matrix consisting of maltodextrin (15% *w*/*v*), β-cyclodextrin (2.5% *w*/*v*), and hydroxypropyl methylcellulose (0.5% *w*/*v*). It ensures comparable structural characteristics across the different formulations. The only varying factor was the encapsulated core material: grapefruit peel extract (ES), pure naringin (NS), and their respective liposomal forms (ELS and NLS).

Across all samples, particles displayed predominantly spherical to quasi-spherical morphology, with sizes ranging approximately from 5 to 10 µm. The surfaces were generally smooth but showed typical spray-drying artefacts such as shrinkage, surface indentations, and wrinkles. This morphology is consistent with previously described spray-dried systems that use carbohydrate-based encapsulation materials [54,55].

Liposomal formulations (ELS and NLS) showed more uniform, rounded, and intact particle structures with fewer surface irregularities. This may be attributed to the presence of liposomal carriers in the matrix, which may improve the structural cohesion and film-forming properties of the droplets during drying. Similar improvements in particle integrity for lipid-containing spray-dried systems have been reported in previous microencapsulation studies [55,56].

Overall, the SEM analysis confirmed that despite minor visual differences related to the core material, all spray-dried samples maintained desirable morphological characteristics suitable for stable dry powder delivery systems.

### 3.4. In Vitro Release Profile in Simulated Gastrointestinal Fluids

**Figure 7:**
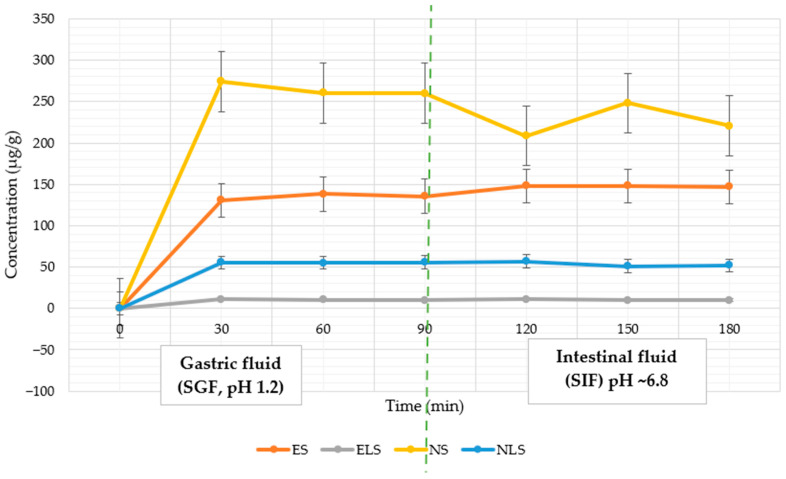
In vitro NR release from spray-dried formulations was evaluated in SGF (pH 1.2) and SIF (pH ~6.8). Results are expressed as mean ± SD (*n* = 3).

It was hypothesised that the application of a dual-encapsulation system consisting of liposomal entrapment and microencapsulation using carbohydrate-based wall materials would provide a modified and sustained release of naringin through simulated gastric (SGF, pH 1.2) and intestinal (SIF, pH ~6.8) fluids compared to single microencapsulation. In total, 500 mg of powder was used in each test, allowing for standardised comparisons between different groups of formulations. Based on the formulation composition, the theoretical NR content in a 500 mg sample was NS—163.82 mg, NLS—6.55 mg, ES—4.53 mg, and ELS—0.94 mg. The result is demonstrated in Figure 7.

#### 3.4.1. NR Release in Gastric Phase (30–90 min)

The release of NR during the gastric phase varied significantly, indicating the influence of the capsule on the kinetics of release. The non-liposomal formulation (NS) exhibited the highest release, reaching 274.26 ± 2.15 µg/mL after 30 min and 260.07 ± 1.73 µg/mL after 90. Despite this high initial release, the overall dissolution efficiency remained low (~5–6% of the theoretical drug content), likely due to rapid saturation of the medium followed by crystal recrystallisation or acid-induced degradation. This behaviour aligns with earlier findings that crystalline flavonoids display poor aqueous solubility and reduced stability in acidic environments due to strong intermolecular hydrogen bonding and high crystal lattice energy [41].

NLS (dual-encapsulated NR) released 55.36 ± 0.44 µg/mL at 30 min and 55.87 ± 0.33 µg/mL at 90 min, corresponding to ~59–60% of its measured aqueous solubility (93.3 µg/mL). This demonstrates that the lipid bilayer and carbohydrate shell act synergistically to limit drug diffusion under acidic conditions, in agreement with previous studies that have shown rigidified liposomal membranes reduce permeability in SGF [53].

For extract-based systems, ES showed rapid release (130.67 ± 3.21 µg/mL at 30 min; 135.71 ± 2.16 µg/mL at 90 min), corresponding to ~94–98% of theoretical content. The high efficiency (91.8%) is likely facilitated by co-extracted polar compounds, such as flavonoid glycosides and organic acids, acting as natural solubilising agents. In contrast, ELS (dual-encapsulated extract) exhibited the lowest absolute release (9.87–10.85 µg/mL), yet this represented ~32–36% of its theoretical loading, indicating that liposomal encapsulation successfully retained NR in the gastric phase, allowing for delayed release into the intestinal stage.

#### 3.4.2. NR Release in Intestinal Phase (120–180 min)

When the medium was changed to simulated intestinal fluid (SIF, pH 6.8), the release of NLS slightly increased to 56.89 ± 0.68 µg/mL after 120 min and remained stable at 52.07 ± 0.55 µg/mL after 180 min. It shows a pH-dependent release pattern where liposome destabilisation and capsule matrix hydration at neutral pH promote controlled diffusion.

NS, which excludes most of its content in SGF, showed lower concentrations in SIF (208.53 ± 1.95 µg/mL at 120 min and 220.82 ± 1.65 µg/mL at 180 min). ELS showed a delayed but sustained release, reaching 11.24 ± 0.41 µg/mL at 120 min and 9.94 ± 0.36 µg/mL at 180 min, confirming gut-mediated disintegration of the carbohydrate matrix. Despite the low absolute concentration, this behaviour supports the concept of site-specific delivery. ES peaked at 148.35 ± 2.37 µg/mL at 120 min, with a minor decline to 146.88 ± 2.01 µg/mL at 180 min, indicating passive diffusion without pH-triggered modulation.

These results confirm that dual-encapsulation systems (NLS, ELS) reduce early gastric release and promote sustained intestinal delivery more effectively than single-encapsulation systems (NS, ES). Similar dual-barrier effects—where a hydrophobic lipid bilayer protects against gastric acid and a carbohydrate shell modulates hydration and erosion—have been reported for other polyphenol delivery systems [56]. The synergistic effect of the double capsule is likely due to the lipid bilayer acting as a hydrophobic barrier, limiting diffusion in acidic environments. At the same time, the carbohydrate matrix inhibits hydration and erosion, while promoting pH-dependent release and penetration into the intestine [57].

## 4. Physical Characterisation of Buccal Films

### 4.1. Visual Appearance of Buccal Films

Buccal films were successfully prepared using the solvent casting method, and four formulations—EP1, EP2, NP1 and NP2—were developed based on different polymer combinations and active ingredients. Formulations EP1 and EP2 contained grapefruit peel extract prepared with 70% ethanol, while NP1 and NP2 were based on a pure naringin solution (50 mg/mL in 70% ethanol). The polymers used in all formulations were hydroxypropyl methylcellulose (HPMC), EP1 and NP1, which contained sodium alginate (ALG), and EP2 and NP2, which contained polyvinyl alcohol (PVA). All films contained glycerol as a plasticiser.

**Figure 8:**
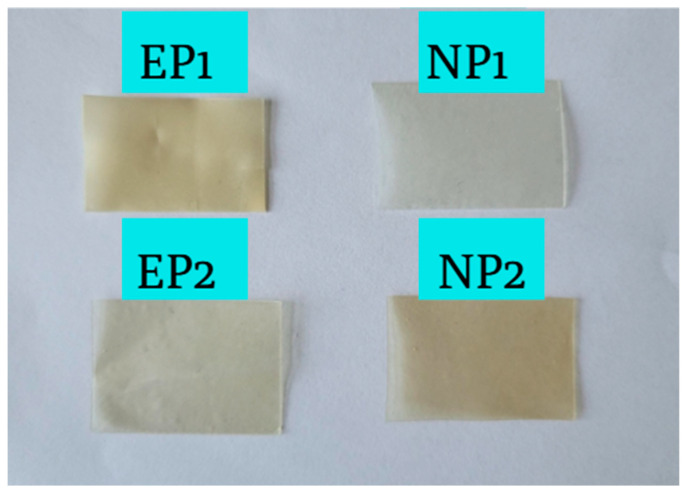
Visual appearance of buccal film samples. EP1 and EP2 (grapefruit peel extract), NP1 and NP2 (pure naringin). EP1/NP1: HPMC + ALG; EP2/NP2: HPMC + PVA.

Visual inspection (Figure 8) revealed that all prepared films were homogeneous, smooth, and free from visible defects, such as air bubbles or cracks, indicating the proper dispersion of the film-forming components. However, apparent differences in transparency and colour were observed between the extract-based (EP1, EP2) and NR-based (NP1, NP2) films. EP1 and EP2 appeared to be light yellow to light brown, which is due to the natural pigmentation of the grapefruit extract, which contains flavonoids and other polyphenolic compounds. It is noteworthy that the surface of EP2 was slightly more transparent and uniform, most likely due to the clarifying properties of the PVA film.

In contrast, NP1 was almost colourless and the most transparent. At the same time, NP2 had a faint yellow tint, possibly due to the interaction of PVA and naringin or due to minor oxidative changes during drying.

These observations suggest that extract-based films typically exhibit higher pigmentation and opacity, which may impact consumer perception and acceptability, while also indicating the presence of multiple bioactive components compared to films containing a single active compound. The choice of secondary polymer (ALG or PVA) also affected the appearance and texture of the film. PVA-based films (EP2, NP2) showed higher visual smoothness and flexibility, likely due to the better film-forming properties of PVA [58]. Meanwhile, ALG-based films (EP1, NP1) were slightly matte and stiffer, which may affect their mucoadhesive properties.

### 4.2. Solubility, Moisture Content, and Mucoadhesive Properties of Buccal Films

The in vitro dissolution performance and mucoadhesive behaviour of the developed buccal films were evaluated using artificial saliva (pH 6.8) and texture analysis. Results are presented in Table 6.

The highest release of naringin in artificial saliva was observed for NP2 (69.97 ± 3.01 µg/mL) and NP1 (63.99 ± 2.64 µg/mL), corresponding to dissolution efficiencies of 40.9% and 37.5%, respectively. These results reflect the efficient matrix hydration and polymer swelling, especially in PVA-based films, which promote faster wetting and better dispersion of molecules [59,60].

In contrast, extract-loaded films (EP1, EP2) released lower absolute amounts of NAR (27.08 ± 1.42 µg/mL and 18.45 ± 1.05 µg/mL, respectively), but achieved moderate dissolution efficiencies relative to their drug loading (40.2% and 26.6%, respectively). Notably, EP1 (ALG-based) exceeded EP2, consistent with the literature, which indicates that alginate matrices promote drug release at near-neutral pH through ion-exchange and gradual erosion mechanisms [61,62].

**Figure 9:**
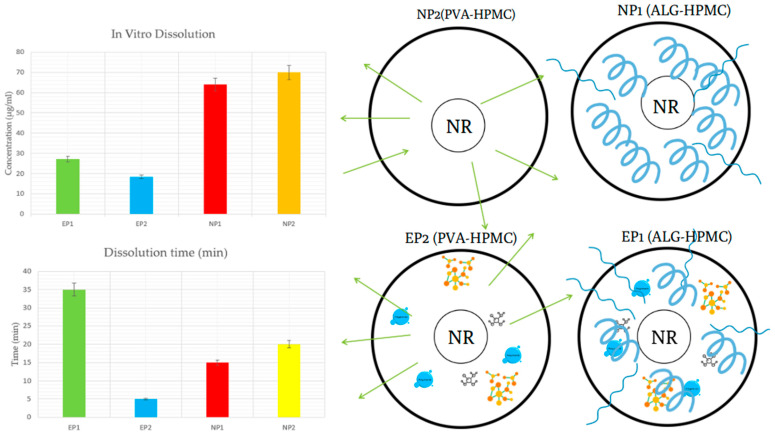
Dissolution time of buccal film formulations (EP1, EP2, NP1, NP2) in artificial saliva (pH 6.8), showing complete film disintegration time. Schematic diagrams illustrate the proposed release mechanisms based on polymer composition and the type of active ingredient. NP2 (PVA–HPMC, pure NR)—rapid hydration and diffusion; NP1 (ALG–HPMC, pure NR)—dense gel matrix restricting diffusion; EP1 (ALG–HPMC, grapefruit peel extract)—improved wettability due to polyphenols, organic acids, pectins, and essential oils; EP2 (PVA–HPMC, grapefruit peel extract)—faster erosion facilitated by co-extracted components but with lower absolute release due to reduced NR loading.

Figure 9 presents these findings along with the complete dissolution time of the films, defined as the time required for the entire buccal film to dissolve fully into artificial saliva. EP1 exhibited the longest dissolution time (~35 min), suggesting a slower matrix erosion rate despite moderate drug release. EP2 shows the fastest dissolution (~5 min), likely due to PVA’s rapid hydration and erosion properties. NP1 and NP2 dissolved more slowly, in 15 and 20 min, respectively, suggesting that variations in polymer structure and the way films absorb water influence the breakdown rate.

Mucoadhesion testing showed that NP2 exhibited the highest adhesion force (0.09 N) and work of adhesion (0.47 N·s), which can be attributed to PVA’s hydrophilicity and ability to form hydrogen bonds with mucin. NP1 also exhibited good adhesion (0.46 N·s), likely due to the ionic interactions of alginate with mucosal surfaces. In contrast, EP1 and EP2 had lower or negative work of adhesion values, suggesting that specific extract components may interfere with optimal polymer–mucin interactions. This may be due to polyphenolic or flavonoid compounds in the extract forming non-specific interactions with mucin or the polymer chains, thus disrupting the formation of stable adhesive networks [60,63]. Additionally, extract constituents may increase interfacial tension or alter surface wettability, reducing effective contact with mucosal surfaces [64]. Interestingly, visual inspection during artificial saliva exposure revealed that all films softened rapidly upon contact with liquid and adhered well to the glass test surface. This suggests that localised swelling and hydration can still promote surface attachment, even when probe-measured mucoadhesion is low.

Moisture content varied among formulations, with extract-based films (EP1, EP2) having higher values (13.48–15.25%), likely due to the hygroscopic nature of sugars and polyphenols in the extract [65]. In comparison, NP1 and NP2 had lower moisture levels (~11.5%), which showed better mechanical stability.

### 4.3. In Vitro Release Profile in Artificial Saliva

**Figure 10:**
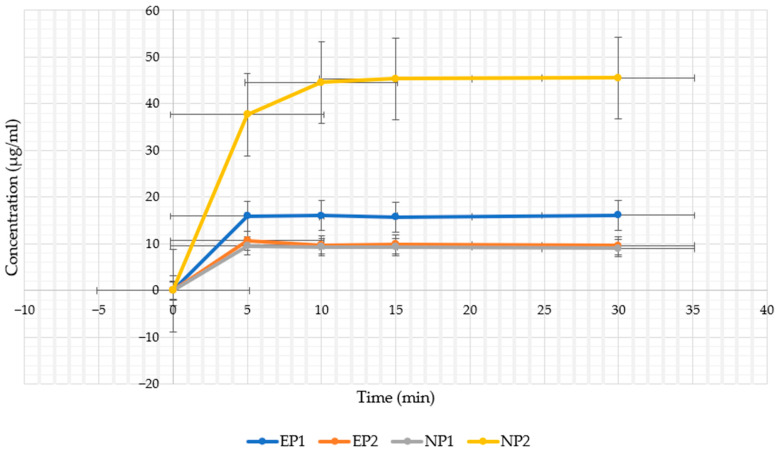
In vitro release profiles of naringin from buccal film formulations (EP1, EP2, NP1, NP2) over 30 min in artificial saliva (pH 6.8). Error bars represent standard deviations (*n* = 3).

The release of NR from extract-based buccal films (EP1 and EP2) was assessed in artificial saliva and is presented alongside pure compound films in Table 6 and Figure 10. Dissolution assay evaluated by the actual maximum release of NR from fully dissolved films (Table 6).

The NP2 formulation exhibited the highest cumulative release, reaching 65.1% within 30 min. These results support the findings of El Sharawy et al. (2017) [65,66] and Elgharbawy et al. (2024) [67], who showed that polyvinyl alcohol (PVA) combined with hydroxypropyl methylcellulose (HPMC) promotes rapid hydration and facilitates diffusion of poorly soluble drugs (e.g., ibuprofen) by forming a highly conductive polymer matrix [31].

The EP1 and EP2 films released approximately 59.4% and 52.1% of their total naringin content, respectively, despite having substantially lower initial loading. This behaviour is consistent with previous reports suggesting that polyphenols and co-extracted phytochemicals can enhance wettability, disrupt polymer–polymer interactions, and accelerate film matrix erosion [68].

In contrast, NP1 showed the lowest release (14.1% in 30 min) despite containing the highest drug content. This reduced release may be related to sodium alginate forming a dense gel barrier in aqueous media, thereby limiting the diffusion of the drug. This reduced release may be attributed to sodium alginate forming a dense gel matrix upon hydration, which restricts drug diffusion, consistent with findings from recent studies using alginate-based buccal systems [62,69].

These findings confirm that both polymer selection (PVA vs. ALG) and the nature of the active ingredient (pure compound vs. plant extract) have a significant influence on buccal film productivity. Extract-based films, although showing lower mucoadhesive properties, appear to be more suited for rapid-release applications. In contrast, PVA–naringin films such as NP2 offer a favourable balance between release kinetics and adhesion.

## 5. Conclusions

In the present study, single microencapsulation of naringin with carbohydrate-based wall materials substantially increased its aqueous solubility compared to the crystalline form, reaching 306.42 µg/mL for the spray-dried powder. This value is markedly higher than the ~30–40 µg/mL reported under standard conditions by Zhang et al. (2015) [70]. Dual encapsulation combining liposomal entrapment with microencapsulation also enhanced solubility relative to pure naringin; however, the values (e.g., 93.32 µg/mL for NLS) were lower than those from single microencapsulation, as the liposomal barrier slowed immediate dissolution while providing greater stability. Buccal films, particularly the PVA–HPMC formulation (NP2), achieved dissolution efficiencies of 40.9% in artificial saliva, consistent with the rapid hydration and diffusion.

Release studies demonstrated that dual encapsulation slowed NR release under gastric conditions and sustained delivery in the intestinal phase, whereas single microencapsulation promoted faster but less controlled release. Among buccal films, NP2 provided the highest total release and mucoadhesive strength, highlighting the role of polymer selection in optimising both drug availability and mucosal retention. Interestingly, grapefruit peel extract-based formulations, despite lower absolute NR content, frequently showed higher relative release efficiencies, likely due to the presence of co-extracted phytochemicals—such as flavonoids, organic acids, and sugars—that improve wettability, matrix hydration, and molecular diffusion.

These results support the initial hypotheses of the study: (i) the double capsule effectively limits release in the acidic gastric environment, supporting intestinal delivery, (ii) PVA-based oral films outperform alginate-based films in terms of release and mucoadhesion, and (iii) grapefruit peel extract increases the relative release of NR compared to the pure compound, likely due to synergistic co-components.

Meanwhile, in vitro findings suggest that dual-encapsulation systems may enhance stability and site-specific release, supporting their potential for improved intestinal absorption and therapeutic efficacy in nutraceutical or pharmaceutical applications.

Although the in vitro results are promising, future in vivo investigations are needed to evaluate the impact of enzymatic degradation, mucosal penetration efficiency, and intestinal absorption mechanisms, which may influence the real-world applicability of these delivery systems.

## Figures and Tables

**Table 1:** The composition of *C. paradisi* peel extract and NR-loaded liposome formulations.

Composition			
Formulation ID *	Lipoid S100	Cholesterol	Total Lipid Phase: Extract or Naringin Ratio
EL1	100 mg	10 mg	1:1
EL2	200 mg	20 mg	2:1
NL1	100 mg	10 mg	1:1
NL2	200 mg	20 mg	2:1

* EL: extract-loaded liposome; NL: NR-loaded liposome. All experiments were conducted in triplicate (*n* = 3).

**Table 2:** Composition of buccal film formulations.

Component	EP1	EP2	NP1	NP2
(HPMC)	12%	12%	12%	12%
(ALG)	2%	–	2%	–
(PVA)	–	2%	–	2%
Glycerol	4%	4%	4%	4%
70% Ethanolic Peel Extract/NR Solution	70%	70%	70%	70%
Purified Water	30%	30%	30%	30%

“–“ indicates that the component was not included in the formulation. Sample IDs: E1, E2, N1, N2. All experiments were conducted in triplicate (*n* = 3).

**Table 3:** Data are presented as mean ± standard deviation (*n* = 3). Letters (a, b, c) indicate statistically significant differences within each column (*p* < 0.05). The formulation ID is listed in Table 1.

Formulation	Size (nm)	PDI	Zeta Potential (mV)
EL1	101.5 ± 5.08 a	0.362 ± 0.018 c	−17.5 ± 0.88 b
EL2	93.93 ± 4.70 b	0.144 ± 0.007 a	−20.3 ± 1.02 bc
NL1	98.57 ± 4.93 ab	0.225 ± 0.011 b	−10.4 ± 0.52 a
NL2	96.96 ± 4.85 ab	0.151 ± 0.017 c	−25.8 ± 1.29 c

**Table 4:** Powder yield, moisture content, encapsulation efficiency (EE%) and aqueous solubility determination of different spray-dried formulations.

Sample ID	Powder Yield (%)	Moisture Content (%)	Encapsulation Efficiency (EE) (%)	Solubility NR (µg/mL)
ES	43.00 ± 2.15	4.12 ± 0.206	90.91 ± 4.54	138.80 ± 4.25 *
ELS	41.05 ± 1.60	3.81 ± 0.19	99.36 ± 4.96 *	17.36 ± 1.01
NS	36.70 ± 1.83	4.21 ± 0.21	81.08 ± 4.05	306.42 ± 10.34 *
NLS	38.15 ± 1.91	5.58 ± 0.279 *	94.60 ± 4.73 *	93.32 ± 6.01

Data are presented as mean ± standard deviation (*n* = 3). * Columns indicate statistically significant differences between groups (*p* < 0.05). Sample ID explanations: ES—Extract without liposomes (spray-dried); ELS—Extract-loaded liposomal powder; NS—Naringin without liposomes (spray-dried); NLS—Naringin-loaded liposomal powder.

**Table 5:** Theoretical and dissolved amounts of naringin (NR) and dissolution efficiency (%) of different spray-dried formulations.

Sample ID	Theoretical Amount of NR (mg)	Dissolved Amount of NR (mg)	Dissolution Efficiency DE (%)
NS	163.82 ± 2.10	9.19 ± 0.31 *	5.6 ± 0.28
NLS	6.55 ± 0.22	2.80 ± 0.18 *	42.7 ± 2.13
ES	4.53 ± 0.15	4.16 ± 0.12	91.8 ± 4.59
ELS	0.94 ± 0.05	0.52 ± 0.03	55.3 ± 2.76

* The difference between the samples’ parameters is statistically significant at *p* * < 0.05.

**Table 6:** In vitro dissolution results, moisture content, and mucoadhesive properties of buccal film formulations (mean ± SD, *n* = 3). Dissolution efficiency is calculated relative to the theoretical maximum drug content in each film.

Sample ID *	In Vitro Dissolution Test (µg/mL)	Dissolution Efficiency (%)	Moisture Content (%)	Peak Force (Adhesiveness N)	Work of Adhesion (N·s)
EP1	27.08 ± 1.42	40.2	13.48 ± 0.45	0.02 ± 0.005	−0.53 ± 0.12
EP2	18.45 ± 1.05	26.6	15.25 ± 0.52	0.07 ± 0.01	−0.25 ± 0.008
NP1	63.99 ± 2.64	37.5	11.46 ± 0.40	0.08 ± 0.01	0.46 ± 0.10
NP2	69.97 ± 3.01	40.9	11.49 ± 0.42	0.09 ± 0.01	0.47 ± 0.11

* Sample ID codes and formulation compositions are listed in Table 2.

## Data Availability

The data presented in this study are available on request from the corresponding author.
